# Chemical set enrichment analysis: Novel insights into sex‐specific alterations in primary metabolites in posttraumatic stress and disturbed sleep

**DOI:** 10.1002/ctm2.511

**Published:** 2021-12-22

**Authors:** Aditi Bhargava, Sili Fan, Callan R. Lujan, Oliver Fiehn, Thomas C. Neylan, Sabra S. Inslicht

**Affiliations:** ^1^ Department of Obstetrics and Gynecology Center for Reproductive Sciences University of California San Francisco San Francisco California USA; ^2^ NIH West Coast Metabolomics Center University of California Davis Genome Center Davis California USA; ^3^ Department of Psychiatry and Behavioral Sciences San Francisco VA Health Care System University of California San Francisco San Francisco California USA

Dear Editor,

We found that women demonstrate more primary metabolite disturbances than men with similar posttraumatic stress disorder (PTSD) severity including a decrease in tryptophan metabolites indoles might be due to gut microbiota dysbiosis. PTSD may develop after exposure to actual or threatened death, serious injury, or sexual violence; women are at a greater risk. The contribution of disordered sleep in PTSD in altering primary metabolites was examined. Primary metabolites regulate several physiological functions, serve as substrates for neurotransmitters, and are altered in psychiatric disorders. Mass spectrometry was used to ascertain metabolites in 90 plasma samples from individuals with chronic PTSD and control subjects. We adjusted for BMI and age in our analyses. Men and women with PTSD did not differ in PTSD severity or history of childhood trauma (Table S1).

Sex aggregated data analysis revealed several metabolites that were significantly altered between control and PTSD groups (Figure S1). Next, sex‐segregated chemical set enrichment analysis^1^ on primary metabolites identified seven and two metabolite nodes altered in women and men with PTSD, respectively, compared with sex‐matched controls (Figure 1A,B). Each node contained two or more metabolites; men and women with PTSD did not share any primary metabolite alterations (Figure 1A,B). Since PTSD symptom presentation is highly variable between individuals,^2^ and, in our cohort, women had significantly greater PCLC scores than men (Table S1), we reasoned that individual PCL‐C measures may associate differently with metabolites (Figure 1C and Table S2). Serine levels were lower in women with PTSD, whereas glycine levels were negatively associated with cluster D symptoms on the PCL‐C, suggesting that with more hyperarousal, glycine levels decreased in women, but not men (Figure 1C).

FIGURE 1Sex differences in primary metabolites in posttraumatic stress disorder (PTSD). The Clinician‐Administered PTSD Scale (CAPS) for DSM‐IV was used to diagnose PTSD and the PTSD checklist‐Civilian version (PCL‐C) determined PTSD severity. Sleep was monitored using laboratory‐based polysomnography and actigraphy. (A) Chemical set enrichment (ChemRICH) analysis of primary metabolites in men and women with PTSD compared with controls after adjusting for BMI and age. ChemRICH is a statistical enrichment approach based on chemical structure similarity/chemical ontologies and is an alternative to pathway analysis that relies on limited biochemical knowledge annotations. It yields study‐specific, non‐overlapping sets of all identified metabolites. Since ChemRICH sets have a self‐contained size, thus *p*‐values do not rely on the size of the background database. Blue nodes contain metabolite clusters that were decreased, purple nodes contain metabolites that were both increased or decreased, and red nodes contain metabolites that were increased in PTSD versus control individuals. Branched‐chain and sulfur‐containing amino acids, and unsaturated fatty acids were increased, indoles and cyclic amino acids were decreased, whereas the non‐polar amino acid node contained metabolites that either increased or decreased in women with PTSD compared to controls. A Venn diagram showing seven nodes were specific to women with PTSD and two nodes were specific to men with PTSD compared with controls. (B) Volcano plots of specific metabolites within each node in men and women with PTSD after correcting for type 1 error and adjusting for BMI and age. (C) Box plots of specific amino acids with the seven nodes that differed between women and two nodes in men with PTSD compared to controls shown in (A). Glycine levels were associated negatively with PCL‐C cluster D in women with PTSD (*r* = –0.47, *p* = 0.036), but not controls, whereas the association of serine level was lost in women with PTSD. Cysteine and 2‐AB levels were elevated in PTSD compared with controls but did not show any significant association with specific clusters of PTSD symptoms. Fructose levels increased in men with PTSD versus controls, but fructose levels were associated negatively with PCL‐C scores. Box plot analysis: Mann–Whitney *U*‐test and *p* < 0.05 considered significant

**Figure:**
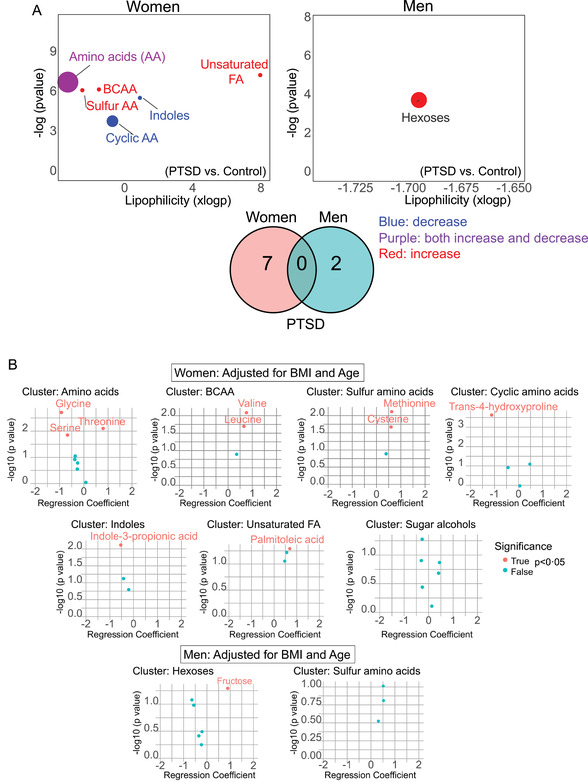

**Figure:**
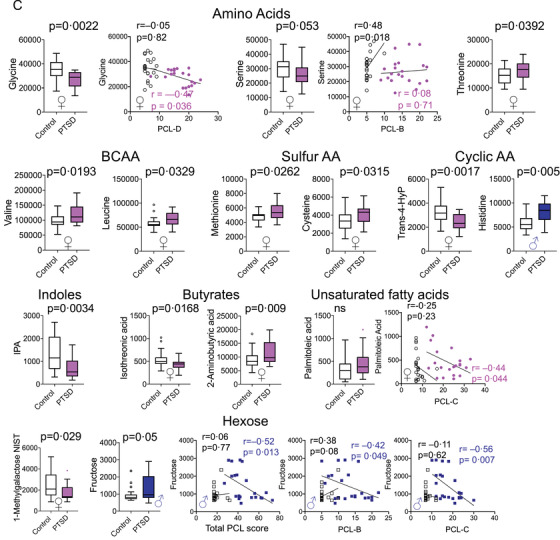

Serine, a neurotransmitter, serves as a precursor for the synthesis of glycine, cysteine, and 2‐aminobutyric acid, butyrate, and is synthesized directly from glucose (Figure 2A). In the human myocardium, 2‐aminobutyric acid increases glutathione levels via activated protein kinas activation to protect against oxidative stress.^3^ An individual's physiological and metabolic state alters glucose metabolism and the generation of non‐essential amino acids. Serine and glycine shuttle between the glia and neurons where glycine induces the release of serine, a coagonist for NMDA receptors. Together they regulate long‐term potentiation^4^ and are critical for the consolidation of extinction of previously conditioned fear memories.^5^ Our data suggest that alterations in subsets of metabolites could be protective in PTSD (Table S2). Changes in metabolite levels in combat veterans with PTSD are reported,^6^, ^7^ but the contribution of sleep or sex has not been investigated.

FIGURE 2Posttraumatic stress disorder (PTSD)‐specific alterations in amino acids with respect to their biosynthesis pathway in humans from glucose. (A) Essential amino acids cannot be synthesized and must be obtained from the diet, whereas non‐essential amino acids can be synthesized from glucose as it enters the tricarboxylic cycle. Essential amino acids are shown in grey boxes, and those metabolites synthesized as a by‐product of the gut microbiome are shown in green boxes. Specific amino acids that were increased are shown in red, decreased in blue (women‐specific outlined in pink and men‐specific in blue). (B) Sex‐specific disturbances in sleep measures. Box plots showing sex‐ and/or PTSD‐specific alterations in total sleep time (in minutes) decreased. Two‐way ANOVA: Sex: ns, PTSD: *p* = 0.004, and Sex X PTSD: ns. Sleep quality worsened, two‐way ANOVA: Sex: ns, PTSD: *p* < 0.0001, Sex X PTSD: ns and log‐transformed delta power sleep were lower in people with PTSD versus controls, two‐way ANOVA: Sex: *p* = 0.007, PTSD: *p* = 0.005, and Sex X PTSD: ns. Greater PTSD symptoms (reflected by the total PCL‐C score) were negatively associated with total sleep time (TST; *r* = –0.41, *p* = 0.005 and *r* = –0.32, *p* = 0.033, women and men, respectively), and Pittsburgh Sleep Quality Index (PSQI) was positively associated with greater PTSD symptoms (*r* = 0.84 and 0.74, *p* < 0.001, women and men, respectively). Delta power was negatively associated with PTSD (*r *= –0.35, *p *= 0.02) in men alone. (C) Linear regression showing association of PCL‐C scores with three different measures of sleep. (D) Sex differences in primary metabolites after accounting for sleep measures. ChemRich analysis of primary metabolites in men and women with PTSD compared with controls after adjusting for BMI, age, and one of the three sleep measures shown (TST (min), PSQI, or log‐transformed delta power (ln(μV2)). Blue nodes contain metabolite clusters that are decreased, purple nodes contain metabolites that are both increased or decreased, and red nodes contain metabolites that are increased in PTSD versus control individuals

**Figure:**
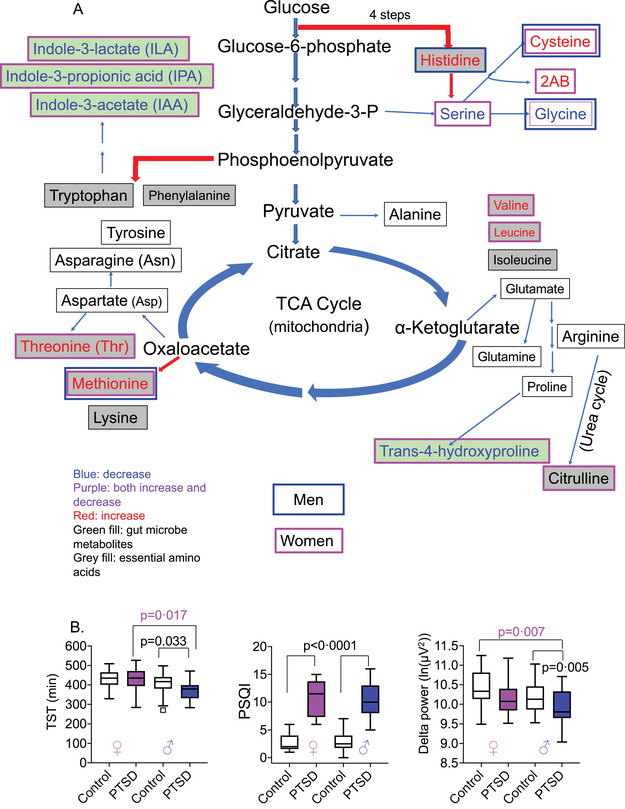

**Figure:**
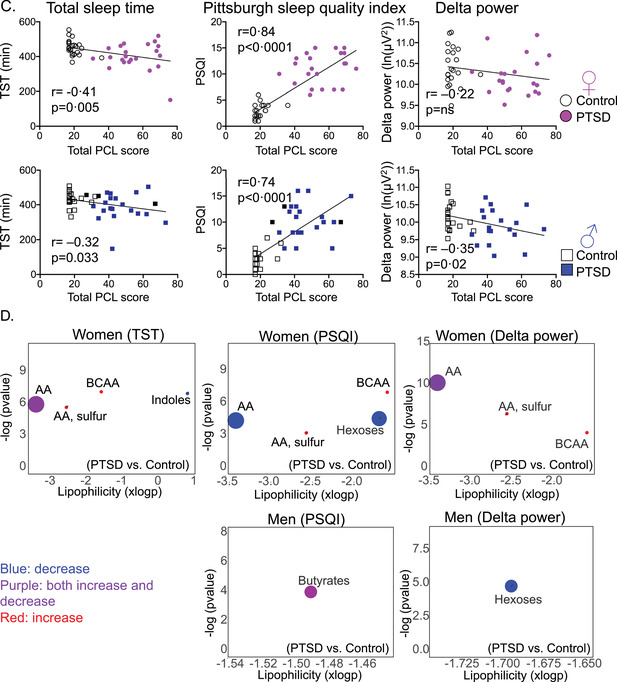

Sleep disturbances are associated with overall poor health.^8^ Both men and women with PTSD had lower total sleep time (TST) on actigraphy and worse self‐reported sleep quality on the Pittsburgh Sleep Quality Index (PSQI) compared with controls (Figure 2B). Delta power, a measure of deep sleep activity, decreased in PTSD and showed a significant sex difference. Men had lower delta power sleep activity than women (Figure 2B). Greater PTSD symptoms were associated with lower TST in both women and men, and PSQI was associated with greater PTSD symptoms in both women and men. Delta power was lower in men with PTSD compared to controls, but not in women (Figure 2C). Humans lack the ability to synthesize eight essential amino acids, including tryptophan, that must be obtained from the diet. They are mostly absorbed by the gut and metabolized by the resident microbiota. Tryptophan is metabolized to a myriad of biologically active compounds by four different pathways (serotonin, tryptamines, kynurenine, and indoles). TST accounted for alterations in all metabolite nodes in men and two of the six metabolite nodes in women (Figures 2D, 3A, and Table S2). When PSQI was included as a confounder, new, non‐overlapping nodes were found to be significant in women and men; indoles, hexose, and amino acid nodes were decreased in women (Figure 2D). Delta power sleep accounted for 50% of the nodes in women (Table S2). Interestingly, higher testosterone levels were associated with lower delta power in men (Figure 3C).

FIGURE 3Sex‐specific contribution of sleep variables on primary metabolites. (A) Venn diagrams to visualize significant metabolites while adjusting for one sleep variable at a time. (B) Linear regression and box plots of various amino acids with sleep variables. Box plots of specific amino acids with individual nodes that differed between women and men with posttraumatic stress disorder (PTSD) compared to controls. (C) Log‐transformed delta power (ln[μV2]) was associated negatively with testosterone in men. (D) Changes in insulin and tryptophan levels in women and men with PTSD. Box plots of tryptophan and albumin levels in women and men. No significant differences were seen in tryptophan and albumin levels in women with PTSD compared with controls, whereas tryptophan levels were significantly elevated in men with PTSD compared with controls (*p* = 0.044; Mann–Whitney *U*‐test). (E) Blood insulin levels were determined after an oral glucose challenge at various times shown. Mixed‐effect analysis showed that insulin levels changed with time (*p* < 0.0001) in women and men, but only differed between PTSD and controls in men (*p* = 0.0136). (F) Tryptophan levels correlated negatively with insulin levels at 60 min in women (*r* = –0.30, *p* = 0.04), but positively in men, although the relationship did not reach statistical significance

**Figure:**
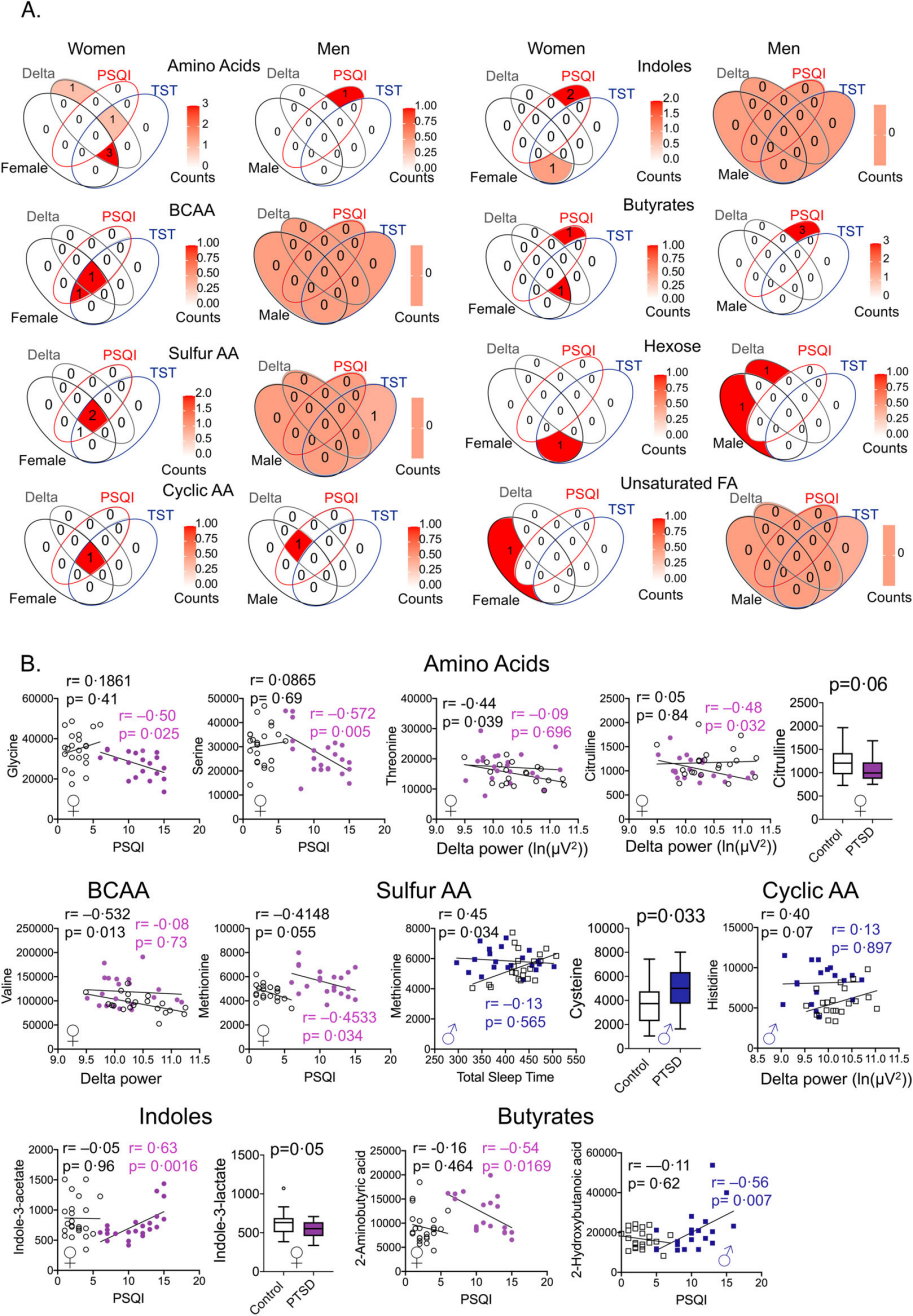

**Figure:**
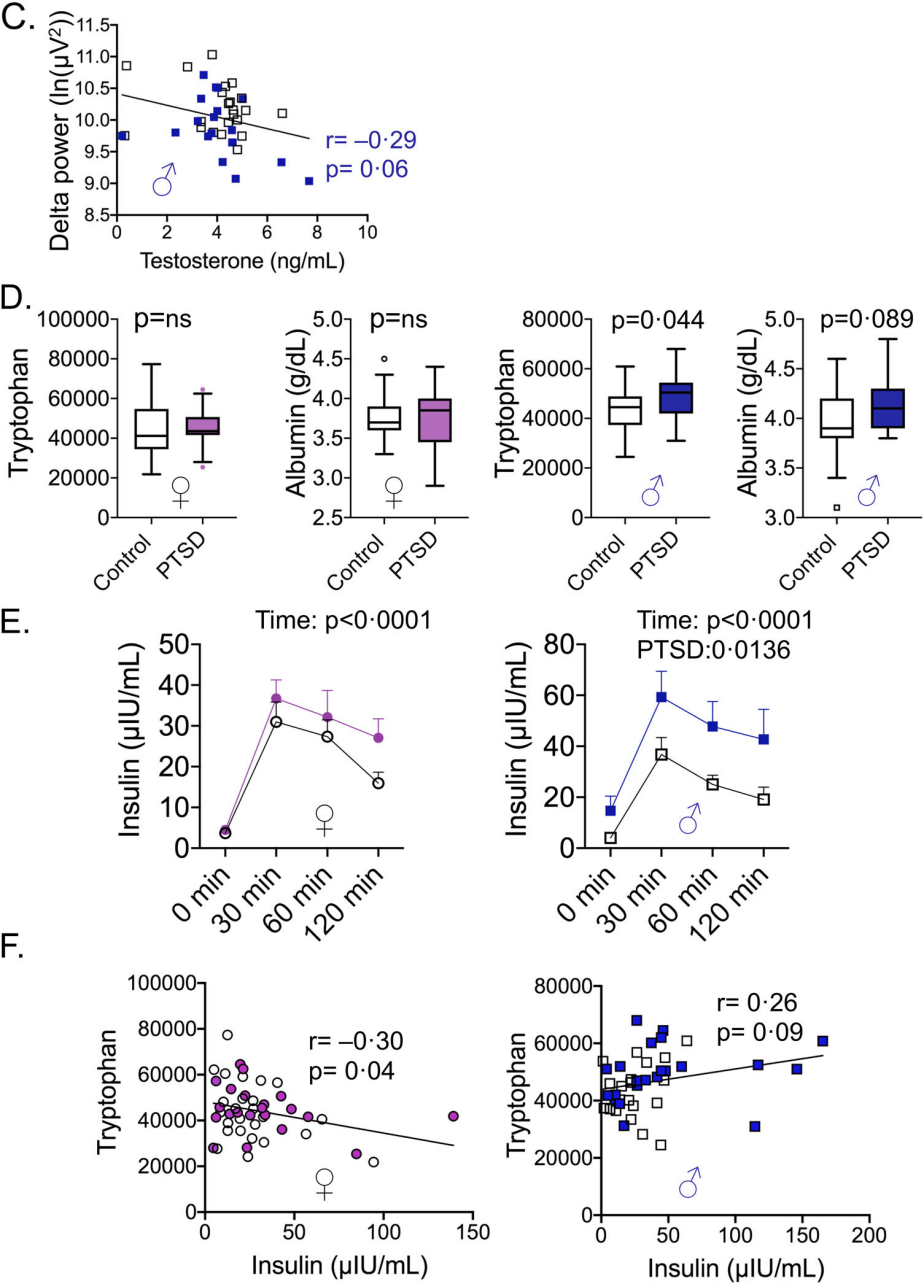

Plasma insulin, albumin, and amino acids levels together modulate the transport of free tryptophan to the brain.^9^ In our cohort, no difference in insulin levels was seen between PTSD and controls in women. The plasma albumin levels did not differ between controls and PTSD in either sex. Insulin levels were significantly elevated in men with PTSD compared with controls (Figure 3D,E). Tryptophan levels were associated negatively with insulin levels in women (Figure 3F) with concomitant increases in levels of several amino acids that compete with a tryptophan carrier for influx into the brain. Increases in palmitoleic acid levels may be compensatory, more so in women than in men, which can potentially displace albumin from tryptophan in order to generate free tryptophan.

Tryptophan is absorbed in the gut and converted by the actions of gut microbiota such as *Lactobacillus* to indoles (Figure 4). While the role of serotonin in mood disorders, anxiety, and other disorders is well known, we show here for the first time that the indole metabolite, indole‐3‐propionic acid, is decreased in women with PTSD. Poorer sleep quality was further associated with decreased levels of two additional indoles, indole‐3‐lactic and acetic acids; the indoles regulate immune and gut barrier functions (Figure 4B), and their decreased levels might contribute to altered immune and gut barrier function in women. Diets rich in butyrates that support the growth of beneficial gut bacteria such as *Lactobacillus* may serve as non‐invasive interventions, especially for women.

**FIGURE 4:**
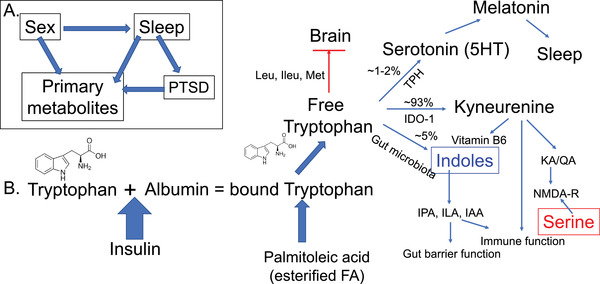
Sex‐specific alterations in the tryptophan metabolism pathway in posttraumatic stress disorder (PTSD). (A) Sex, sleep, and PTSD alter primary metabolites. (B) Albumin‐bound tryptophan is present in the circulation and dynamic increases in insulin promote binding of albumin to tryptophan, whereas esterified fatty acids can displace tryptophan from albumin. Free tryptophan is then transported to the brain by a transport carrier. Several amino acids, such as leucine, valine, and so forth compete with tryptophan for binding to the transport carrier, which can decrease the influx of free tryptophan into the brain. Reduced free tryptophan levels in the brain can influence the production of serotonin and melatonin, affecting brain function and sleep. In the gut, tryptophan is converted to indoles by the action of microbes such as *Lactobacillus*. These indoles have a protective effect on the gut barrier and immune functions. Serine can serve as an NMDA receptor agonist and alter neuronal function. Thus, disturbances at multiple levels in the tryptophan pathway may contribute to the pathogenesis of PTSD

Surprisingly, most sex steroid metabolites did not differ between controls and PTSD in either men or women (Table S3), nor did sex steroids associate with PTSD. Testosterone levels were ∼10‐fold higher in men (Table S3).

In conclusion, there was no overlap in PTSD‐related alterations in men and women in any primary metabolite nodes or individual amino acids within those nodes and associated pathways. A balance between levels of essential amino acids, insulin, and albumin determines the availability and transport of free tryptophan to the brain, which might in turn influence the production of serotonin and melatonin. Distinct cellular pathways operate within functional clusters in men and women (Figure 4).

## CONFLICT OF INTEREST

The authors declare that they have no conflict of interest. The views, opinions and/or findings contained in this research are those of the authors and do not necessarily reflect the views of the Department of Defense, Department of Veteran Affairs, or NIH and should not be construed as an official DoD/Army/VA/NIH position, policy, or decision unless so designated by official documentation. No official endorsement should be made.

### AUTHOR CONTRIBUTIONS

Aditi Bhargava, Thomas C. Neylan, and Sabra S. Inslicht contributed to the design of the study. Aditi Bhargava wrote the first draft of the manuscript with input from Sabra S. Inslicht. Aditi Bhargava, Sili Fan, Oliver Fiehn, Thomas C. Neylan, and Sabra S. Inslicht revised the final manuscript and all authors reviewed the final version. Callan R. Lujan and Sabra S. Inslicht collected patient data. Sili Fan, Oliver Fiehn, Sabra S. Inslicht, and Aditi Bhargava extracted and analyzed the data.

## Supporting information



Supporting information

FigureS1

## References

[1] Barupal DK , Fiehn O . Chemical similarity enrichment analysis (ChemRICH) as alternative to biochemical pathway mapping for metabolomic datasets. Sci Rep. 2017;7:14567. 29109515 10.1038/s41598-017-15231-w PMC5673929

[2] Galatzer‐Levy IR , Bryant RA . 636,120 ways to have posttraumatic stress disorder. Perspect Psychol Sci. 2013;8:651‐662. 26173229 10.1177/1745691613504115

[3] Irino Y , Toh R , Nagao M , et al. 2‐Aminobutyric acid modulates glutathione homeostasis in the myocardium. Sci Rep. 2016;6:36749. 27827456 10.1038/srep36749 PMC5101505

[4] Neame S , Safory H , Radzishevsky I , et al. The NMDA receptor activation by d‐serine and glycine is controlled by an astrocytic Phgdh‐dependent serine shuttle. Proc Natl Acad Sci U S A. 2019;116:20736‐20742. 31548413 10.1073/pnas.1909458116 PMC6789919

[5] Davis M . NMDA receptors and fear extinction: implications for cognitive behavioral therapy. Dialogues Clin Neurosci 2011;13:463‐474. 22275851 10.31887/DCNS.2011.13.4/mdavis PMC3263393

[6] Mellon SH , Bersani FS , Lindqvist D , et al. Metabolomic analysis of male combat veterans with post traumatic stress disorder. PLoS One. 2019;14:e0213839. 30883584 10.1371/journal.pone.0213839 PMC6422302

[7] Somvanshi PR , Mellon SH , Flory JD , et al. Mechanistic inferences on metabolic dysfunction in posttraumatic stress disorder from an integrated model and multiomic analysis: role of glucocorticoid receptor sensitivity. Am J Physiol Endocrinol Metab. 2019;317:E879‐E898. 31322414 10.1152/ajpendo.00065.2019 PMC6879860

[8] Richards A , Metzler TJ , Ruoff LM , et al. Sex differences in objective measures of sleep in post‐traumatic stress disorder and healthy control subjects. J Sleep Res. 2013;22:679‐687. 23763708 10.1111/jsr.12064 PMC3958933

[9] Daniel PM , Love ER , Moorhouse SR , , Pratt OE . The effect of insulin upon the influx of tryptophan into the brain of the rabbit. J Physiol. 1981;312:551‐562. 7021801 10.1113/jphysiol.1981.sp013643 PMC1275568

