# Supplementary material for: Chemical set enrichment analysis: Novel insights into sex‐specific alterations in primary metabolites in posttraumatic stress and disturbed sleep
Source: Clin Transl Med. 2021 Dec 22;11(12):e511. doi: 10.1002/ctm2.511 (PMC8694503; doi:10.1002/ctm2.511)
Supplement: Supplementary file 1 — Supporting information [file CTM2-11-e511-s001.docx]

**Chemical set enrichment analysis: novel insights into sex-specific alterations in primary metabolites in posttraumatic stress and disturbed sleep**

Aditi Bhargava^1^ PhD*, Sili Fan^2^, Callan Lujan^3^, Oliver Fiehn^2^ PhD, Thomas C. Neylan^3^ MD, and Sabra S. Inslicht^3^ PhD*.

**Affiliations:**

^1^Center for Reproductive Sciences, Department of Obstetrics and Gynecology, University of California San Francisco, CA 94143, USA.

^2^NIH West Coast Metabolomics Center, University of California Davis Genome Center, Davis, CA 95616, USA.

^3^UCSF Department of Psychiatry and Behavioral Sciences, San Francisco VA Health Care System, 4150 Clement St. (116P), San Francisco, CA 94121, USA.

*To whom correspondence should be addressed:

Aditi Bhargava, PhD

Email: [Aditi.bhargava@ucsf.edu](mailto:Aditi.bhargava@ucsf.edu)

and

Sabra S. Inslicht, PhD

**Running Title: Sex-specific primary metabolites in PTSD**

**Key Words:** Amino acids; ChemRich plot; Delta power sleep; insulin; mass spectrometry; PSQI.

**List of Abbreviations**

AAs: amino acids

BMI: body mass index

ChemRICH: Chemical Set Enrichment Analysis

CAPS:Clinician-Administered PTSD Scale

COVID-19: Coronavirus disease-19

DSM: Diagnostic and Statistical Manual of Mental Disorders

EEG: electroencephalogram

EOG: electrooculograms

GCRC: General Clinical Research Center

GC-TOF-MS: gas chromatography /time of flight mass spectrometry (GC-TOF MS)

HPLC: High pressure liquid chromatography

HPA: hypothalamic-pituitary-adrenal

ICU: Intensive care unit

KYN: Kynurenine

NREM: non-rapid eye movement

NMDA: *N*-methyl-D-aspartate receptors

OGTT: Oral Glucose Tolerance Test

PTSD: posttraumatic stress disorder

PSQI: Pittsburgh Sleep Quality Index

TST: Total sleep time

**Supplemental materials and methods**

**Human subjects**

This study was a 2 x 2 cross-sectional design with 4 groups (PTSD/control × women/men) of 44 individuals with current chronic PTSD and 46 control subjects. Participant’s age ranged from 19-39 years. Data from 4 participants were excluded due to difficulties in blood collection. Sleep measures were recorded in an inpatient sleep laboratory at the General Clinical Research Center (GCRC) at the University of California, San Francisco. The Committee on Human Research at the University of California, San Francisco approved this study. Written informed consent was provided to all participants before enrollment and start of any study procedures.

PTSD subjects met DSM-IV criteria as ascertained using the Clinician-Administered PTSD Scale (CAPS) or score >40. Control subjects had no lifetime or current history of a PTSD diagnosis. Women participants were premenopausal, and were scheduled during the follicular phase of the menstrual cycle. All study procedures were timed according to habitual sleep onset, determined by actigraphy and sleep diary in the week prior to the GCRC study. Study participants were limited to one cup of caffeine daily, maintained regular bed and waking times, did not consume alcohol, and were drug-free. Exclusion criteria: history of traumatic brain injury, presence of neurologic disorders or systemic illness; use of psychiatric, anticonvulsant, antihypertensive, sympathomimetic, steroidal, statin or other prescription medications; obesity (defined as body mass index (BMI) >30); alcohol abuse or dependence in the prior two years; substance abuse or dependence in the previous year; any psychiatric disorder with psychotic features; bipolar disorder or obsessive-compulsive disorder; and pregnancy. Exclusion criteria for control subjects also included a lifetime history of major depressive disorder or panic disorder.

**Psychiatric diagnoses and trauma history**

The Life Stressor Checklist-Revised interview was used to determine trauma exposure and age of occurrence.^1^ The PTSD Checklist (civilian version) for DSM-IV (PCL-C)^2^ was used as a self-reported measure of severity of chronic PTSD symptoms.^3^ The PCL-C consists of 17 items that correspond to the DSM-IV criteria and include intrusive thoughts and re-experiencing symptoms (cluster B), avoidance (cluster C), and hyperarousal (cluster D). The structured clinical interview for DSM-IV, non-patient edition (SCID-NP) was used to diagnose all other psychiatric disorders, including major depressive disorder.^4^

**Sleep clinic and measures**

The Pittsburgh Sleep Quality Index^5^ was used to provide a subjective assessment of sleep quality, sleep latency, sleep duration, sleep efficiency, sleep disturbances (including nightmares), use of sleep medication, and daytime dysfunction over the previous month as described elsewhere.^6^

**Polysomnographic measurements**

Ambulatory polysomnography (Nihon Kohden Trackit Ambulatory Recording System) was used to record electroencephalogram (EEG) at leads C3, C4, O1 and O2, left and right electrooculograms (EOG), submental electromyogram, bilateral anterior tibialis EMGs, and electrocardiogram in accordance with standardized guidelines by Rechtschaffen.^7^ Electrode impedance was set at < 5K Ohm at the start of the recording. The EEG and EOG leads were referenced to linked mastoids. Raw EEG signals were filtered and amplified, then digitized at 256 Hz and recorded to a removable hard disk in European Data Format file format. The low frequency and high frequency hardware filters on the recorder were single pole analog filters with 3 db points at 0·5 Hz and 100 Hz. Pass Plus was utilized for both visual scoring and quantitative EEG analysis of the digitized polysomnography data.

**Power spectral analysis for polysomnographic measures**

Pass Plus (Delta Software) analytic software was used to measure sleep activity in all frequency bands delta through gamma from the C3 electrode by power spectral analysis. The C4 electrode was used if there was excessive artifact. A limitation of Pass Plus is that artifact removal is accomplished by removal of whole epochs tagged with artifact. This has the potential to introduce additional confounds given the removal of typically longer bouts of uncontaminated EEG. Therefore, epochs were tagged for slow and fast artifact for additional analyses. Primary analyses were conducted with all epochs and then checked for the impact of removal of epochs with slow and fast artifact. Removal of fast artifact (for bandwidths alpha and above) and slow artifact (for bandwidths delta and theta) did not significantly impact our findings in non-rapid eye movement (NREM) sleep. All results are therefore reported without removal of epochs containing artifact. Pass Plus applied a 5μV smoothing constant to eliminate spurious waves caused by electrical jitter. Power spectral analysis was conducted on all epochs of NREM and REM sleep. Epochs visually scored as wake were not included in these analyses. Visual scoring was conducted by a highly experienced registered polysomnography technician, blind to PTSD status, classified all 30-second epochs in every sleep record as wake; stages 1, 2, 3; REM; or movement using current American Academy of Sleep Medicine criteria. Total sleep time was defined by time spent in epochs scored as NREM stages 1 through 3 and stage REM. Pass Plus was used to perform Fast Fourier Transformation analysis on 4·0 second Welch tapered windows with 2 second overlap, yielding 15 windows per 30-second epoch. Power spectra for delta (1-4 Hz) were analyzed to address our hypothesis with respect to delta spectral power. Delta sleep spectral power density (μV2) was natural log transformed to normalize its distributions.

**Blood collection and clinical laboratory measures**

Blood was collected at habitual wake-up time on the morning after the second night on the GCRC, while the subject was fasting, from an indwelling catheter inserted the night before. Blood (10mL) was drawn into a chilled EDTA tube and processed for plasma separation for mass spectrometry analysis.

**Oral glucose tolerance test (OGTT)**

Oral Glucose Tolerance Test (OGTT) was performed on the morning of day two after a 10-hour overnight fast. Subjects were given 75g dextrose in 250 cc of water, and blood samples were taken at baseline and 30, 60 and 120 minutes for measurement of glucose and insulin levels. Glucose was measured by the hexokinase method (Roche Applied Sciences, Indianapolis, IN) on the Cobas C501, at the Washington University Core Laboratory. Insulin measurement was performed by EMD Millipore Corporation (St. Charles, MO, USA) by radioimmunoassay.

**Analysis of primary metabolites in human plasma using gas chromatography /time of flight mass spectrometry (GC-TOF MS)**

Primary metabolites that included sugar phosphates, amino acids, hydroxyl acids, and free fatty acids, were measured using GC-TOF MS. Briefly, metabolites from plasma were extracted using 1 milliliter of degassed, −20°C cold solvent mixture of acetonitrile (ACN):isopropanol (IPA):water (H_2_O) (3:3:2, v/v/v) per 20 µL sample aliquot. Samples were vortexed for 10 seconds, shaken for 5 min and then centrifuged for 2 min at 12,800 x g. Two 450 µL supernatant aliquots were transferred to new tubes. To remove any excess protein and triacylglycerides, the supernatant evaporated and resuspended in 500 µL 1:1 acetonitrile:water and vortexed for 10 seconds, centrifuged for 2 min at 12,800 x g. The supernatant was transferred to a clean tube and then dried down in a CentriVap concentrator. Samples were derivatized with 10 μL of methoxyamine hydrochloride (20mg/mL) in pyridine and subsequently by 90 μL of MSTFA for trimethylsilylation of acidic protons, including a mixture of C8–C30 fatty acid methyl esters as internal standards for retention index correction.^8^ Internal standards in GC-MS included retention index markers for database annotations^9^. These internal standards are not used for quantification. Instead, we use mixtures of 30 external standards (Quality Control (QC) mix) in dilution curves before and after each batch of samples, in addition the highest-concentrated QC mix sample after each set of 10 biological samples, again, as described elsewhere.^9^

**Data acquisition, extraction, and processing of metabolites**

Data were acquired using the following chromatographic parameters^10^: Column: Restek corporation Rtx-5Sil MS (30 m length x 0.25 mm internal diameter with 0·25μm film made of 95% dimethyl/5%diphenylpolysiloxane); Mobile phase: Helium; Column temperature: 50-330°C Flow-rate: 1 mL min-1; Injection volume: 0·5μL; Injection: 25 splitless time into a multi-baffled glass liner Injection temperature: 50°C ramped to 250°C by 12°C s-1; Oven temperature program: 50°C for 1 min, then ramped at 20°C min-1 to 330°C, held constant for 5 min. Data processing*.* Raw data files were preprocessed directly after data acquisition and stored as ChromaTOF-specific *.peg files, as generic *.txt result files and additionally as generic ANDI MS *.cdf files. ChromaTOF vs. 4·0 was used for data preprocessing without smoothing, 3 s peak width, baseline subtraction just above the noise level, and automatic mass spectral deconvolution and peak detection at signal/noise levels of 5:1 throughout the chromatogram. Apex masses are reported for use in the BinBase algorithm. Result *.txt files were exported to a data server with absolute spectra intensities and further processed by a filtering algorithm implemented in the metabolomics BinBase database. Given compound was identified and cross-referenced with external database identifiers such as InChI key, PubChem ID, and KEGG ID. Following equation was then used for normalizations for metabolite *i* of sample *j*:

$$metabilite ij normalized=\frac{metaboliteij raw}{mTICj}.mTICaverage$$

The normalized data are shown as peak heights for the quantification ion (mz value) at the specific retention index. Missing values were imputed using half of the minimum detected value for each compound. Auto-scaling was performed to make compounds the same scale.^11^

All metabolomic data were investigated using pooled quality control samples and blank samples. Metabolites that had more than 30% relative standard deviation in pooled QC samples were removed, and metabolites that showed less than 3-fold higher intensity compared to blank samples were removed as well. We do not count such signals as genuine metabolites; hence, the final data sheet is what was used for statistical analyses. Briefly, the number of reported signals from raw processed data to final reported data went from 313 metabolites to 266 metabolites. Technical errors reduce biological power, so findings reported in this work were observed at statistical power despite possible technical variance.

**Statistical analysis**

We first analyzed data in a sex aggregated manner and found several metabolites to be significantly altered between control and PTSD groups (Fig. S1). Men with PTSD were more likely to be obese than other groups. Therefore, we adjusted for BMI and age in our analyses. We next performed our more detailed analyses in a sex segregated manner. We conducted robust linear regression for each sex (i.e. male and female, respectively) to examine the association between compound intensity, PTSD status, sleeping factors, and to examine the interaction effect between sex and PTSD status for each metabolite. Specifically, robust linear regression was conducted with compound intensity as response and PTSD status as predictor, controlling for body mass index and age. The effect size was determined by the coefficient of the regression model, with a positive value indicating compound intensity being higher in PTSD compared with control and a negative value indicating the opposite. We used the Benjamini Hochberg procedure to correct for multiple comparisons. The p-values and effect sizes from robust linear regression were used as input for the ChemRICH analysis^12^ to study the enrichment effect on metabolite clustered defined according to chemical similarity. Kolmogorov–Smirnov test was used to calculate the p-value for each of the metabolite clusters. We further used Venn-diagrams to highlight the sex difference from the ChemRICH analyses. Volcano plots were used to visualize the significance and effect sizes of each metabolite in each of the significant clusters for each sex after correcting for type 1 errors. We also performed the same analysis but with each of the three sleeping factors (total sleep time (min), PSQI, and log-transformed delta energy (μV2sec)) as confounding variables. We used box plot and Venn diagrams to visualize the significance effect while adjusting for sleep variables. We used the statistical computing language R version 3.6.3. Robust linear regression was performed in MASS library.

**References**

1. Wolfe J, Kimerling R, Brown PJ, Chresman KR, Levin K. Psychometric review of the life stressor checklist-revised. Lutherville, MD: Sidran Press; 1996.

2. Blanchard EB, Jones-Alexander J, Buckley TC, Forneris CA. Psychometric properties of the PTSD Checklist (PCL). *Behaviour Research & Therapy* 1996; **34**(8).

3. Blake DD, Weathers FW, Nagy LM, et al. The development of a Clinician-Administered PTSD Scale. *J Trauma Stress* 1995; **8**(1): 75-90.

4. Spitzer RL, Williams JB, Gibbon M, First MB. The Structured Clinical Interview for DSM-III--R (SCID) : I. History, rationale, and description. *Archives of general psychiatry* 1992; **49**(8): 624-9.

5. Buysse DJ, Reynolds CF, 3rd, Monk TH, Berman SR, Kupfer DJ. The Pittsburgh Sleep Quality Index: a new instrument for psychiatric practice and research. *Psychiatry Res* 1989; **28**(2): 193-213.

6. Richards A, Metzler TJ, Ruoff LM, et al. Sex differences in objective measures of sleep in post-traumatic stress disorder and healthy control subjects. *Journal of sleep research* 2013; **22**(6): 679-87.

7. Kales A, Rechtschaffen A. A manual of standardized terminology, techniques and scoring system for sleep stages of human subjects. : Washington, DC : United States Government Printing Office, 1968.; 1968.

8. La Frano MR, Carmichael SL, Ma C, et al. Impact of post-collection freezing delay on the reliability of serum metabolomics in samples reflecting the California mid-term pregnancy biobank. *Metabolomics* 2018; **14**(11): 151.

9. Fiehn O. Metabolomics by Gas Chromatography-Mass Spectrometry: Combined Targeted and Untargeted Profiling. *Curr Protoc Mol Biol* 2016; **114**: 30 4 1- 4 2.

10. Fiehn O, Wohlgemuth G, Scholz M, et al. Quality control for plant metabolomics: reporting MSI-compliant studies. *Plant J* 2008; **53**(4): 691-704.

11. Wanichthanarak K, Fan S, Grapov D, Barupal DK, Fiehn O. Metabox: A Toolbox for Metabolomic Data Analysis, Interpretation and Integrative Exploration. *PLoS One* 2017; **12**(1): e0171046.

12. Barupal DK, Fiehn O. Chemical Similarity Enrichment Analysis (ChemRICH) as alternative to biochemical pathway mapping for metabolomic datasets. *Sci Rep* 2017; **7**(1): 14567.

**Table S1. Demographics data and clinical characteristics of PTSD and control subjects**.

|  | Men | | Women | |  |
| --- | --- | --- | --- | --- | --- |
| **Variable** | Control (N=22) | PTSD+ (N=22) | Control (N=24) | PTSD+ (N=22) | Total (N=90) |
| Age (Mean ± SD) ^a^ | 30·2 ± 8·76 | 30·6 ± 7·61 | 30·0 ± 7·47 | 30·2 ± 6·82 | 30·3 ± 7·31 |
| Education (Years) (Mean ± SD) ^a^ | 15·5 ± 2·08 | 14·4 ± 2·32 | 15·5 ± 1·92 | 15·3 ± 2·03 | 15·2 ± 2·11 |
| **Race** ^b^ |  |  |  |  |  |
| African American | 1 (4·5%) | 3 (13·6%) | 0 (0·0%) | 2 (9·1%) | 6 (6·7%) |
| Asian/Hawaiian/Pacific Islander | 4 (18·2%) | 1 (4·5%) | 3 (12·5%) | 2 (9·1%) | 10 (11·1%) |
| Caucasian | 17 (77·3%) | 13 (59·1%) | 19 (79·2%) | 14 (63·6%) | 54 (63·5%) |
| Other/Unknown | 0 (0·0%) | 5 (22·7%) | 2 (8·3%) | 4 (18·2%) | 11 (12·2%) |
| Hispanic Ethnicity ^b,c^ | 0 (0·0%) | 5 (22·7%) | 3 (12·5%) | 1 (4·5%) | 9 (10·0%) |
| Current CAPS score ^d^ (Mean ± SD) | 0·0 ± 0·0 | 51·9 ± 13·03 | 0·0 ± 0·0 | 55·2 ± 21·58 | 53·5 ± 17·70 |
| PTSD Symptom Checklist (PCL; Mean ± SD) ^e^ | 19·09 ± 4·21 | 46·0 ± 12·29* | 19·9 ± 4·08 | 55·6 ± 12·27* | 34·8 ± 18·39 |
| Current MDD ^f^ | 0 (0·0%) | 5 (22·7%) | 0 (0·0%) | 3 (13·6%) | 8 (8·9%) |
| Childhood trauma ≤14 years of age^g,h^ | 0 (0·0%) | 8 (36·4%) | 0 (0·0%) | 11 (50·0%) | 19 (21·1%) |
| Hormonal birth control ^b,i^ | NA | NA | 2 (8·3%) | 6 (27·3%) | 8 (17·4%) |
| **BMI** ^b^ |  |  |  |  |  |
| Underweight = <18·5 | 2 (9·1%) | 0 (0·0%) | 1 (4·2%) | 0 (0·0%) | 3 (3·3%) |
| Normal weight = 18·5–24·9 | 10 (45·5%) | 4 (18·2%) | 12 (50·0%) | 13 (59·1%) | 39 (43·3%) |
| Overweight = 25–29·9 | 10 (45·5%) | 5 (22·7%) | 8 (33·3%) | 7 (31·8%) | 30 (33·3%) |
| Obesity = BMI of 30+ | 0 (0·0%) | 13 (59·1%) | 3 (12·5%) | 2 (9·1%) | 18 (20·0%) |
| Smoker ^b, j^ | 3 (13·6%) | 4 (18·2%) | 6 (25·0%) | 6 (27·3%) | 19 (21·1%) |

a based on F-test

b based on Chi-square test

c Three subjects endorsed Hispanic ethnicity but did not select a racial descriptor. Six additional subjects endorsed Hispanic ethnicity, in addition to a racial category of Caucasian or African-American race yielding a total of 9 subjects self-identifying as Hispanic in this sample. Comparison of Hispanic ethnicity, *p*=·063.

d Control subjects had CAPS scores of zero or had an absence of criterion A events. Comparison of male and female PTSD subjects on current CAPS score, *p*=0·54.

e PTSD group by gender interaction on current PCL score, *p*<.05. *:Comparison of PTSD groups vs controls, p<.001. Comparison of male vs female PTSD groups vs controls, *p*<0·01.

f Absence of current MDD was required for inclusion into the control group. Comparison of male and female PTSD subjects on rate of current MDD, *p*=0·43.

g Childhood trauma exposure was defined, based on findings from our prior research, by exposure to 2 or more categories of childhood trauma under the age of 14. Three (6·5%) control subjects reported a history of 1 category of childhood trauma.

h Chi-square test compared frequency of childhood trauma between male and female PTSD subjects only, *p*=·36.

i Chi-square test compared use of hormonal birth control female PTSD and control subjects only, *p*=·09

j based on diary

**Table S2. Sex steroid metabolite concentrations in plasma of men and women**.

| Metabolite | LOD (ng/mL) | Control ± SD (ng/mL)  Women | PTSD ± SD (ng/mL)  Women | Control ± SD (ng/mL)  Men | PTSD ± SD (ng/mL)  Men |
| --- | --- | --- | --- | --- | --- |
| Progesterone (P4) | 0.393 | 0·59 ± 1·19 | <LOD (<0·28) | <LOD (<0·09) | <LOD (<0·09) |
| Testosterone* | 0.144 | 0·55 ± 0·90 | 0·44 ± 0·68 | 4·03 ± 1·39 | 4·17 ± 1·61 |
| Estrone (E1) | 2.702 | <LOD (<0·11) | <LOD (<0·24) | <LOD (<0·16) | <LOD (<0·11) |
| Estradiol (E2) | 0.680 | <LOD (<0·03) | <LOD (<0·06) | <LOD (<0·02) | <LOD (<0·03) |
| Estriol (E3) | 2.882 | 5·7 ± 8·93 | 3·23 ± 2·26 | 3·39 ± 3·96 | 4·68 ± 9·69 |

Steroid metabolite values plasma in ng/mL. Limit of detection (LOD) for each analyte is shown. Values in red text were below LOD. *: p<0·0001 Men vs. Women (sex); PTSD: ns; PTSD x Sex: ns

**Table S3**. Altered primary metabolite clusters in PTSD with sleep confounders

| Men  PTSD | Men  Delta Power | Men  PSQI | Men  TST | Women  PTSD | Women  Delta Power | Women  PSQI | Women  TST |
| --- | --- | --- | --- | --- | --- | --- | --- |
| **Amino Acids (AA)** | | | | | | | |
| . | . | Gly | . | Glycine (Gly) | Gly | . | Gly |
| . | . | . | . | Threonine (Thr) | Thr | . | Thr |
| . | . | . | . | Serine (Ser) | Ser | . | Ser |
| . | . | . | . | . | Citrulline | . | . |
| . | . | . | . | . | β-Ala | β-Alanine | β-Ala |
| **Branched-chain amino acids (BCAA)** | | | | | | | |
| . | . | . | . | Valine (Val) | Val | Val | Val |
| . | . | . | . | Leucine (Leu) | . | Leu | Leu |
| **Sulfur amino acids** | | | | | | | |
| . | . | . | Met | Methionine (Met) | Met | Met | . |
| . | . | . | Cys | Cysteine (Cys) | Cys | Cyst | . |
| **Cyclic amino acids** | | | | | | | |
| Histidine (His) | His | . | . | . | . | . | . |
| . | . | . | 4-Hyp | Trans-4-hydroxyproline (4-Hyp) | 4-Hyp | 4-Hyp | . |
| **Indoles** (Tryptophan metabolites) | | | | | | | |
| . | . | . | . | Indole-3-propionic acid (IPA) | . | . | IPA |
| . | . | . | . | . | . | Indole-3-lactate (ILA) | . |
| . | . | . | . | . | . | Indole-3-acetate (IAA) | . |
| **Butyrates** (Gut microbes) | | | | | | | |
| . | . | Threonic acid | . | . | . | . | . |
| . | . | 3-Hydroxybutyric acid | . | . | . | . | . |
| . | . | 2-Hydroxybutanoic acid | . | . | . | . | . |
| . | . | . | . | Isothreonic acid (ITA) | ITA | . | ITA |
| . | . | . | . | . | . | 2-Aminobutyric acid (2AB) | . |
| **Hexose** | | | | | | | |
| Fructose | . | . | . | . | . | . | . |
| . | Sucrose | . | . | . | . | . | . |
| . | . | . | . | 1-Methylgalactose NIST | . | . | 1-MG NIST |
| **Unsaturated fatty acids (UFA)** | | | | | | | |
| . | . | . | . | Palmitoleic acid | . | . | . |

**Supplementary Figure Legend**

**Fig. S1. Sex aggregated analysis of primary metabolites**. There were seven primary metabolites that were significantly different between PTSD and control subjects when data were analyzed in a sex aggregated manner. Eight metabolites that have not been thoroughly characterized yet, were also identified. P values as shown using Mann-Whitney U-test.
