# Supplementary figures and images for: Chemical set enrichment analysis: Novel insights into sex‐specific alterations in primary metabolites in posttraumatic stress and disturbed sleep
Source: Clin Transl Med. 2021 Dec 22;11(12):e511. doi: 10.1002/ctm2.511 (PMC8694503; doi:10.1002/ctm2.511)

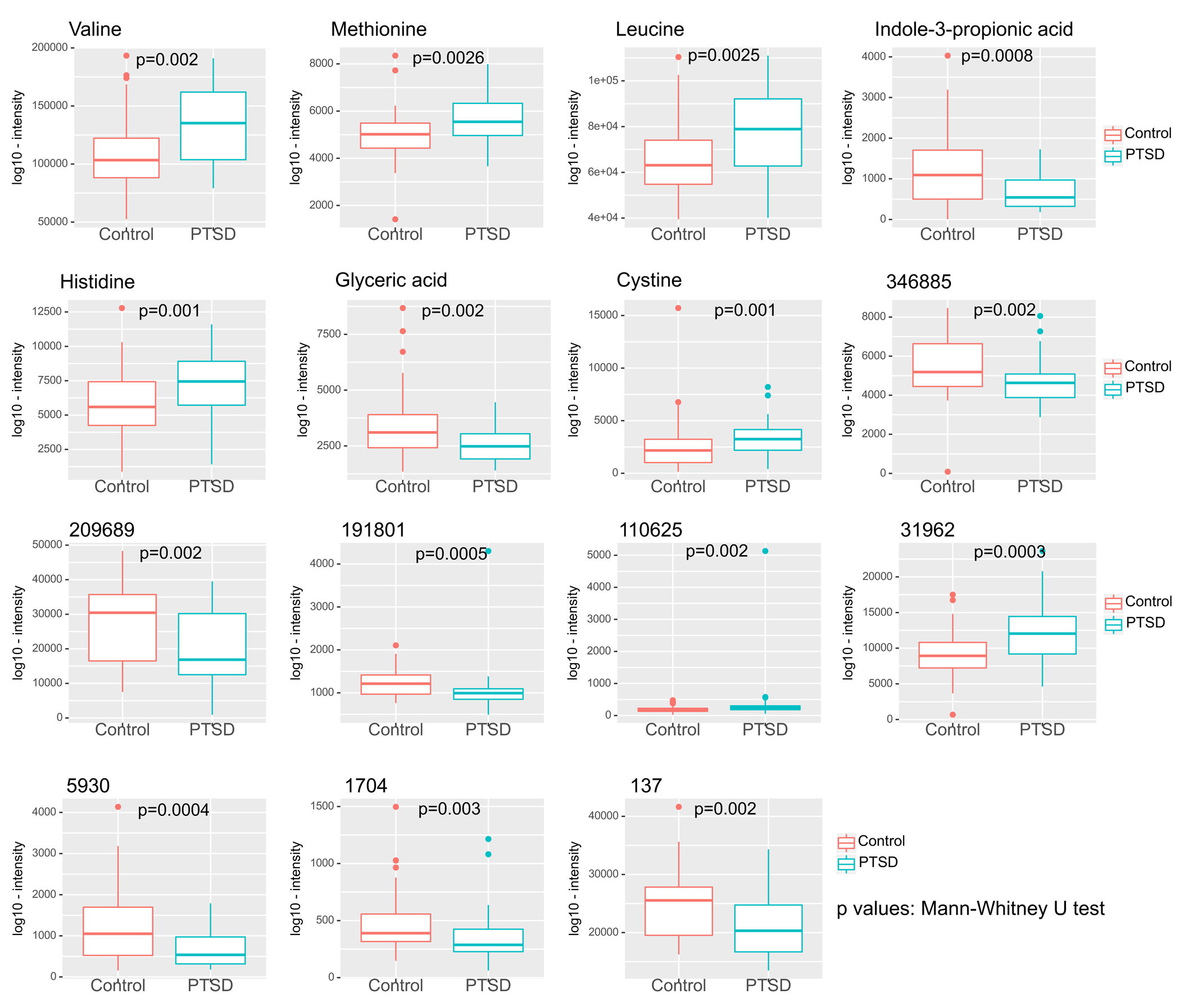

Supplement: Supplementary file 2 — FigureS1 [file CTM2-11-e511-s002.tif]
